# Adenosine A2A receptors in the nucleus accumbens regulate stress resilience and predict susceptibility to stress-induced affective disturbances

**DOI:** 10.1007/s11302-025-10118-2

**Published:** 2026-01-14

**Authors:** Laura Menegatti Bevilacqua, Francisco da Silveira Neto, Axel Fogaça Rosado, Cibele Martins Pinho, Nicolle Platt, Manuella P. Kaster

**Affiliations:** 1 Center for Biological Sciences, Department of Biochemistry, Federal University of Santa Catarina, Florianopolis, Santa Catarina Brazil; 2 Department of Psychiatry and Neuroscience, Université Laval, CERVO Brain Research Center, Quebec City, QC Canada; 3 Department of Psychiatry, Yale University, New Haven, CT USA; 4 ICM, Paris Brain Institute, Hôpital de la Pitié-Salpêtrière, Sorbonne Université, INSERM, CNRS, Paris, France

**Keywords:** Adenosine A2A receptors, Caffeine, Nucleus accumbens, Stress resilience, Synaptic proteins

## Abstract

Individual variability in stress responses contributes to susceptibility or resilience to mood and anxiety disorders. The adenosinergic system, particularly A1 and A2A receptors, modulates synaptic activity in brain regions involved in emotional regulation, including the nucleus accumbens (NAc), a central hub of reward processing and stress responsivity. Despite high adenosine receptor expression in the NAc, their specific role in stress resilience remains poorly understood. Here, we investigated the effects of 10 days of chronic social defeat stress (CSDS) on A1 and A2A receptor expression and synaptic proteins in the NAc of male C57BL/6 mice categorized as resilient (RES) or susceptible (SS) based on their sociability. SS mice exhibited social avoidance and elevated anxiety-like behaviors, whereas RES mice behaved comparably to controls. Neurochemical analyses revealed that RES mice had lower A2A receptor mRNA levels and decreased PSD-95 expression, suggesting reduced excitatory synaptic tone, while A1 receptor and gephyrin levels remained unchanged. To assess whether baseline adenosine signaling predicts stress vulnerability, we applied a caffeine responsiveness index. Caffeine, a non-selective adenosine receptor antagonist (7.5 mg/kg, i.p.), was used to classify Swiss mice as caffeine-responsive or non-responsive based on its psychostimulant effects, which are primarily attributed to A2A receptor antagonism. Following a 14-day washout, animals were exposed to chronic variable stress (CVS), and caffeine-responsive mice showed deficits in motivational and exploratory behaviors. Together, these findings indicate that lower A2A receptor signaling in the NAc supports stress resilience, whereas heightened baseline caffeine sensitivity may predict vulnerability to stress-induced affective disturbances.

**Graphical Abstract:**

Reduced adenosine A2A receptor signaling in the nucleus accumbens (NAc) supports stress resilience, whereas heightened caffeine sensitivity predicts vulnerability. Under chronic social defeat stress, resilient mice display downregulated *Adora2a* mRNA and reduced PSD-95 levels, consistent with attenuated excitatory drive, while susceptible mice maintain A2A receptor activity and show anxiety-like behaviors. In the chronic variable stress paradigm, baseline caffeine responsiveness stratifies outcomes, with responsive mice presenting anxiety- and depressive-like behaviors, whereas mice there are non-responsive to caffeine exhibit disinhibited exploration but preserved motivation. Together, these findings identify baseline adenosine receptor signaling in the NAc as a key determinant of individual responses to chronic stress.

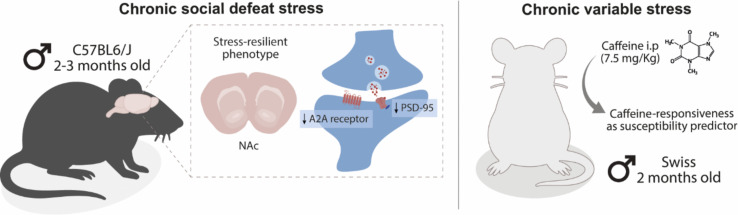

## Introduction

Chronic stress is a major environmental risk factor for psychiatric disorders, which are leading causes of disability, reduced quality of life, and social impairment [1–3]. Proper regulation of brain circuits involved in motivation and reward is essential for adaptive responses to stress. In this sense, the nucleus accumbens (NAc), a central structure in these circuits, integrates glutamatergic inputs from the prefrontal cortex, hippocampus, and amygdala with dopaminergic signals from the ventral tegmental area, enabling the processing of reward, aversive, and emotional information [4]. Its neuronal composition is predominantly GABAergic medium spiny neurons (MSNs) expressing either D1 or D2 dopamine receptors, modulated by smaller populations of GABAergic and cholinergic interneurons [5]. Dysregulation of NAc circuits under chronic stress contributes to motivational and affective impairments and has been implicated in the pathophysiology of multiple psychiatric disorders [6, 7]. Yet, individual responses to stress vary considerably: while some develop debilitating psychopathology, others display resilience, the capacity to maintain or rapidly restore emotional and physiological balance despite adversity [8].

Chronic stress induces multi-layered changes in the NAc across synaptic, molecular, and circuit levels, affecting both excitatory and inhibitory signaling. At the circuit level, chronic social defeat stress (CSDS) enhances hippocampal input to the NAc in stress-susceptible (SS) mice, suggesting that increased excitatory drive may contribute to maladaptive responses [9]. In parallel, CSDS reduces the expression of key inhibitory synapse markers in the NAc, leading to a decreased frequency of miniature inhibitory postsynaptic currents (mIPSCs) in medium spiny neurons (MSNs) of SS animals. These synaptic alterations are associated with social avoidance behaviors and are mirrored in postmortem NAc tissue from individuals with major depressive disorder (MDD) [10]. Additionally, repeated activation of D2-MSNs in mice promotes social avoidance following subthreshold social defeat, highlighting the critical role of D2-MSN activity in mediating vulnerability to stress [11].

Adenosine receptors, particularly the A1 and A2A, are abundantly expressed in the NAc, where they play key modulatory roles in synaptic transmission, neuronal excitability, and reward-related behaviors [12, 13]. A1 receptors are broadly expressed and typically exert inhibitory effects by reducing neurotransmitter release and neuronal firing. In contrast, A2A receptors are primarily localized to D2-MSNs and modulate dopaminergic and glutamatergic signaling, often exerting excitatory effects [14]. Dysregulation of adenosine receptor signaling in the NAc has been implicated in mood disorders and addiction, making these receptors potential targets for therapeutic intervention [15].

In humans, chronic treatment with A2A receptors antagonists, such as istradefylline, has shown improvements in depressive symptoms in patients with Parkinson’s disease [16, 17]. Moreover, polymorphisms in the *Adora2a* gene are classified as anxiogenic and are associated with increased glutamatergic transmission in anxiety and depressive disorders [18–21]. In rodent models, chronic treatment with caffeine, a non-selective A2A receptor antagonist, reverses social avoidance in animals susceptible to CSDS [22]. We previously demonstrated that chronic stress induces increase in hippocampal A2A receptors, an effect associated with cognitive impairment and depressive-like behaviors [23]. Complementarily, A2A receptor knock-out animals show protective effects against stressors, exhibiting higher sociability and reduced anhedonic behaviors and sensitivity to social novelty after CSDS [24–26]. Therefore, it is proposed that the adenosinergic system may contribute to the heterogeneity of behavioral responses to stressors [27–29].

Given that the NAc is a key site of convergence for stress responses and adenosine signaling, the present study evaluated how individual differences in stress vulnerability affect adenosine receptor expression and synaptic proteins, and how these changes relate to behavioral outcomes by comparing A1 and A2A receptor dynamics in RES vs SS mice. These findings provide mechanistic insight into the contribution of NAc adenosine signaling to stress resilience and susceptibility, with potential implications for the pathophysiology of mood and anxiety disorders.

## Methods

### Animals

For the CSDS protocol, male C57BL/6 mice (3–4 months old) were used as experimental subjects, while male Swiss mice (4–6 months old) served as aggressors (AGG). The chronic variable stress (CVS) protocol was conducted using male Swiss mice (2 months old). All animals were group-housed (8 animals per cage) in standard cages (41 × 34 × 16 cm) at the Animal Facility of the Department of Biochemistry, Federal University of Santa Catarina (UFSC), under controlled temperature (21 ± 2 °C), a 12/12 h light/dark cycle (lights on at 7:00 a.m.), with ad libitum access to food (NUVITAL—Nuvilab CR1) and filtered water. Mice were obtained from the Central Animal Facility of UFSC, and all procedures followed institutional ethical guidelines (protocol number: 5290231120).

### Chronic social defeat stress (CSDS)

The CSDS protocol was used to induce chronic psychosocial stress and distinguish stress-SS from RES phenotypes. Swiss mice were individually housed in standard cages (30 × 20 × 13 cm) for at least one month prior to CSDS to establish territorial behavior. Aggressiveness was evaluated over three consecutive daily screening sessions, during which each Swiss mouse was exposed to a novel male C57BL/6 mouse for 3 min. Mice were selected as aggressors (AGG) if they displayed an attack latency under 60 s or performed more than 10 attacks in at least two sessions. Attacks were defined as direct, overtly aggressive behaviors initiated by the AGG. The CSDS protocol consisted of 10 consecutive days of stress exposure. Each day, a C57BL/6 experimental mouse was placed into the home cage of a different AGG for 10 min of physical confrontation, followed by 24 h of sensory contact through a perforated acrylic divider. This setup allowed continued visual, olfactory, and auditory interaction while preventing physical aggression [30]. Coping behaviors were assessed during the physical encounters on days 1, 5, and 10. Active responses (e.g., escape, avoidance, defense) and passive responses (e.g., freezing, submissive posture) were recorded, and an active behavior index was calculated as the number of active minus passive behaviors, normalized by the total number of attacks [31]. Control animals were housed in pairs, separated by a perforated acrylic divider to allow sensory but not physical contact. Pairings were rotated daily to prevent the formation of stable social hierarchies.

### Behavioral assessments

Twenty-four hours after the final CSDS session, experimental animals were subjected to the social interaction (SI) test to determine SS or RES phenotypes, and emotional-like behaviors were evaluated. All behavioral experiments were performed under indirect light conditions (30 lx). Behavioral assessments included the SI test, open field test (OFT), and elevated plus maze test (EPM), which were analyzed using the automated system ANY-maze® (Stoelting Co., USA). The tail suspension test (TST), sucrose splash test (SST), and analysis of coping behaviors were scored manually by an experienced researcher blinded to the experimental conditions.

#### Social interaction test (SI)

The SI was used to evaluate sociability and stress-related avoidance following CSDS exposure. The test exploits the natural exploratory tendencies of mice and their interest in conspecifics. The apparatus consists of a modified open field arena (42 cm^3^) equipped with a perforated acrylic cylinder (10 cm diameter, 15 cm height) fixed to one wall, allowing for sensory but not physical contact with an unfamiliar AGG. Two spatial zones were delineated for analysis: the interaction zone (24 × 14 cm), directly adjacent to the cylinder, and two corner zones (9 × 9 cm each) located in the corners farthest from the cylinder. The SI test consisted of two 150-s phases. In the no-AGG phase, the experimental C57BL/6 mouse freely explored the empty arena. The mouse was then removed, and an unfamiliar AGG was placed inside the acrylic cylinder. In the AGG phase, the experimental mouse was reintroduced for an additional 150 s of exploration. Time spent in the interaction zone and corner zones was recorded, and the SI ratio was calculated by dividing the time spent in the interaction zone during the AGG phase by the time spent in the same zone during the no-AGG phase. Animals with a ratio ≥ 1 were classified as RES, while those with a ratio < 1 were considered SS [30].

#### Open field test (OFT)

The OFT is used to assess locomotor activity and anxiety-like behavior in rodents. The apparatus consists of an acrylic box (42 cm^3^), and it is assumed that anxious animals spend more time in the corners of the arena. Animals were exposed to the open field for 5 min and allowed to explore freely. The total distance traveled was used as a locomotor parameter, while the number of entries and time spent in the central zone (21 cm^2^) were evaluated as anxiety-related measures [32].

#### Elevated plus maze test (EPM)

The EPM was used to assess anxiety-like behavior. The apparatus consisted of two open arms (1 cm high) and two closed arms (30 cm high), arranged in a plus shape and connected by a central platform (10 cm^2^). The test is based on rodents' natural aversion to open, elevated areas and preference for enclosed spaces, creating a conflict between exploration and anxiety-related avoidance. Reduced exploration of the open arms is interpreted as increased anxiety-like behavior. Animals were placed in the center of the maze facing an open arm and allowed to explore freely for 5 min. The number of entries and time spent in each arm (defined by all four paws entering an arm) were recorded, along with risk assessment behaviors, defined as the animal extending its head and forepaws into an open arm while keeping the hind limbs in the central platform or closed arm [33].

#### Sucrose splash test (SST)

The SST was conducted to assess motivational behavior and anhedonia/self-care. The test consisted of spraying a 10% sucrose solution three times on the back of the mouse in an empty and clean cage. Grooming bouts were defined as ≥ 2 s of continuous grooming. The frequency, latency, and interbout interval (time elapsed between successive grooming bouts) of self-grooming were recorded for 10 min [34].

### Transcriptional profiling

Mice were decapitated one hour after behavioral testing, and the NAc was dissected and preserved in 1 mL of TRIzol® reagent (Sigma-Aldrich) for gene expression analysis. Tissues were homogenized and centrifuged at 12,000 x g for 10 min at 4 °C immediately after collection, and supernatants were stored at − 20 °C until RNA extraction. Total RNA was quantified by spectrophotometry (NanoVue Plus™, Biochrom®), and samples with a 260/280 ratio > 1.8 were selected. RNA integrity was confirmed by 0.8% agarose gel electrophoresis stained with ethidium bromide. cDNA was synthesized from 1 μg of total RNA using qScript® cDNA SuperMix (QuantaBio®) in 20 μL reactions and stored at − 20 °C. RT-qPCR was performed in triplicate using TaqMan™ Fast Advanced MasterMix and gene-specific TaqMan probes: *Adora1* (Mm0138023_m1) and *Adora2a* (Mm00802075_m1, FAM-labeled), with *Gapdh* (Mm99999915_g1, VIC-labeled) as the endogenous control. Reactions were run on a StepOnePlus™ Real-Time PCR System (Applied Biosystems®) under the following conditions: 50 °C for 2 min, 95 °C for 20 s, followed by 40 cycles of 95 °C for 1 s and 60 °C for 20 s. Gene expression was analyzed using the 2-^ΔΔCt^ method.

### Protein immunodetection by western blotting

Mice were decapitated one hour after behavioral testing, and the NAc was immediately dissected and frozen in liquid nitrogen until protein extraction. Proteins were extracted by mechanical homogenization in 300 μL of RIPA buffer. Homogenates were centrifuged, and 50 μL aliquots were used for protein quantification via the Lowry method [35]. Protein (60 μg) was separated on 10% sodium dodecyl sulfate–polyacrylamide gel electrophoresis (SDS-PAGE) and transferred to nitrocellulose membranes using a semi-dry system. Membranes were blocked with 5% bovine serum albumin (BSA) in Tris-buffered saline (TBS), washed with TBS containing 0.1% Tween-20 (TBS-T), and incubated overnight at 4 °C with primary antibodies in 2% BSA/TBS-T: A1 adenosine receptor (Invitrogen, 1:1000), A2A adenosine receptor (Santa Cruz, 1:500), β-actin (Cell Signaling, 1:4000), gephyrin (Invitrogen, 1:1000), and postsynaptic density protein 95 (PSD-95; Cell Signaling, 1:1000). After washing, membranes were incubated with horseradish peroxidase (HRP)–conjugated secondary antibodies for 1 h at room temperature and revealed using the Super ECL kit (GE Healthcare) on a Chemi-Doc system (Bio-Rad, LAMEB/UFSC). Immunoreactive bands were quantified by chemiluminescence, and protein levels were expressed as the optical density ratio to β-actin, normalized to controls.

### Caffeine responsiveness to chronic variable stress (CVS)

A separate cohort of male Swiss mice was used to assess caffeine-induced locomotor responses. Baseline locomotor activity was recorded in the OFT for 5 min. Twenty-four hours later, animals received an intraperitoneal (i.p.) injection of caffeine (7.5 mg/kg; Sigma-Aldrich®) and were re-exposed to the OFT for 5 min, 30 min post-injection [36]. The responsiveness index was calculated as the ratio between post-caffeine and baseline total distance traveled. Mice with an index ≥ 1.0 were classified as caffeine-responsive (R), and those with < 1.0 as non-responsive (NR). The CVS protocol was applied by exposing the mice to one stressor per day for 21 consecutive days, including foot shock (0.5 mA, 2 min), restraint stress (2 h), and tail suspension (6 min). Control mice remained in their home cages in groups of five and were handled for 2 min per day [37].

### Statistical analysis

Outliers were identified and removed using Grubbs test, and analyses were conducted in GraphPad Prism 10.6. Normality of distributions was assessed with the Shapiro–Wilk test to determine the appropriate statistical approach. For normally distributed data, repeated-measures ANOVA was used for within-subject comparisons, and one-way and two-way ANOVA with Tukey’s post hoc test was applied for group comparisons. Non-normally distributed data were analyzed using the Kruskal–Wallis test with Dunn’s post hoc test for independent group comparisons, or the Wilcoxon matched-pairs test for measurements within the same subjects (e.g., pre- vs. post-stress values). Linear regression was used to assess the relationship between *Adora2a* expression and sociability. Behavioral z-scores were summarized in radar plots generated in Excel, and data are presented as individual values, mean ± SEM, with statistical significance set at p < 0.05.

## Results

### Behavioral profiling reveals social deficits and anxiety-like phenotype following CSDS

**Fig. 1:**
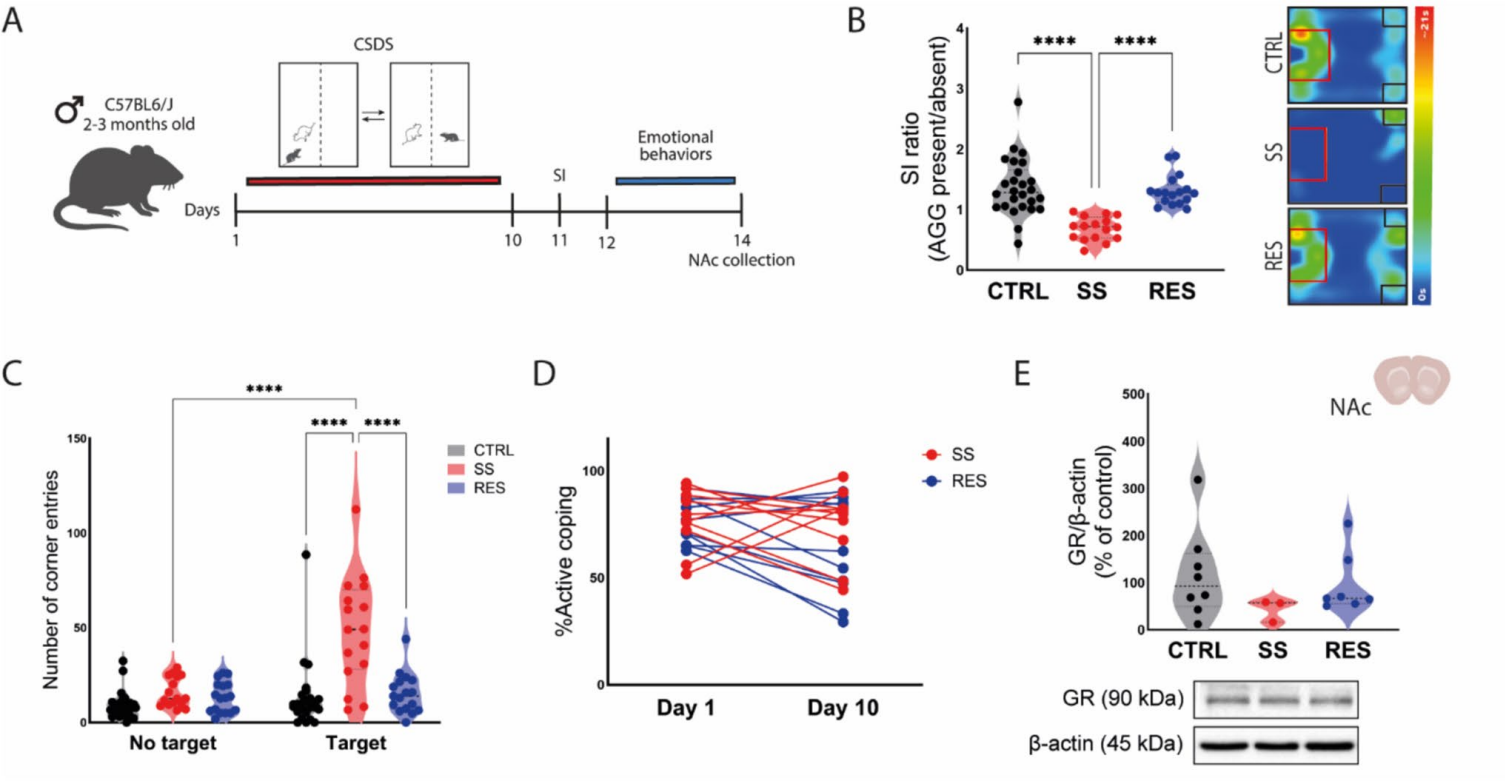
Chronic social defeat stress (CSDS) induces sociability impairments without affecting coping strategies. **A.** Experimental timeline of the CSDS paradigm, followed by social interaction (SI) testing, emotional behavior assessments, and nucleus accumbens (NAc) tissue collection. **B.** SI ratio (time spent in the interaction zone with aggressor/AGG present vs. absent) reveals significant social avoidance in a subset of mice classified as susceptible (SS). Resilient mice (RES) displayed a SI index comparable to that of non-stressed controls (CTRL). **C**. Number of entries into the corner zones during the SI test was increased in SS mice in the presence of the AGG. **D.** Percentage of active coping, measured by the number of attacks endured on day 1 and day 10 of CSDS remained stable across groups. **E.** Glucocorticoid receptor (GR) protein expression in the NAc, shown as a percentage of CTRL levels. Kruskal–Wallis followed by Dunn’s test (SI ratio and GR protein expression), one-way ANOVA followed by Tukey’s *post-hoc* test (corner entries), and two-way repeated measures ANOVA (active coping). Data are shown as individual values, mean ± SEM, ****p < 0.0001

To investigate the behavioral consequences of CSDS, mice were subjected to 10 consecutive days of social defeat, followed by a social interaction (SI) test and emotional behavior assessments (Fig. 1A). Based on their performance in the SI test, animals were classified as either stress-susceptible (SS) or resilient (RES). SS mice exhibited a lower SI ratio compared to both CTRL and RES groups, indicating pronounced social avoidance (Fig. 1B). Consistent with this phenotype, SS mice showed a markedly increased number of corner entries during the SI test in the presence of the AGG, a behavioral indicator of social withdrawal and avoidance (Fig. 1C). In contrast, no differences were observed in coping strategies during the CSDS sessions, as measured by the percentage of active coping across days 1 and 10 (Fig. 1D); both SS and RES groups exhibited comparable levels of exposure to aggression. Finally, no alterations in GR protein expression were observed in the NAc across experimental groups, suggesting that CSDS-induced behavioral phenotypes occurred independently of GR modulation in this region (Fig. 1E).

**Fig. 2:**
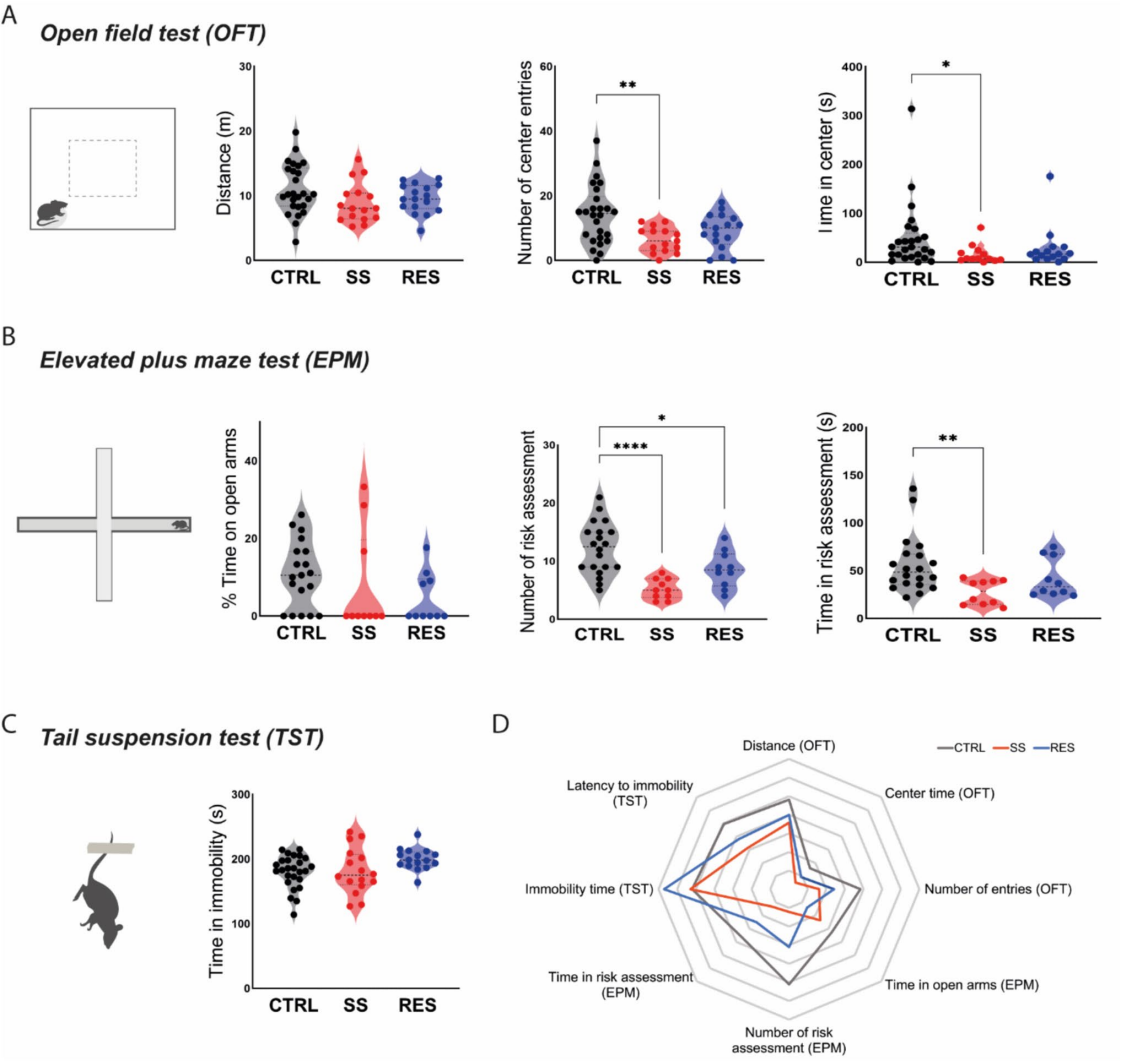
Chronic social defeat stress (CSDS) induces an anxiety-like phenotype in stress-susceptible (SS) mice. **A.** Although the distance traveled was the same between groups (left), SS mice made fewer center entries (middle) and spent less time in the center (right) in the open field test (OFT). **B.** On the elevated plus maze (EPM), percentage of time spent in open arms (left) was also not altered, but number of risk assessments (middle) showed an effect of both CSDS groups, while only SS mice showed impairment in the time spent in risk assessment behaviors (right). **C.** No alterations were observed in the tail suspension test (TST). **D.** Radar plot summarizing behavioral outcomes across all tests. Each axis represents a behavioral measure from OFT, EPM, or TST. Stress SS mice (red) show a distinct behavioral profile compared to control (CTRL, black) and stress-resilient (RES, blue) groups, particularly in parameters related to anxiety. One-way ANOVA followed by Tukey’s *post-hoc* test (OFT: distance; EPM: number of risk assessment; TST: immobility time), and Kruskal–Wallis followed by Dunn’s *post-hoc* test (OFT: center entries and time; EPM: time in open arms and in risk assessment). Data are shown as mean. *p < 0.05, **p < 0.01, ***p < 0.0001

To confirm the emotional impact of CSDS, we assessed locomotor activity, anxiety-like behaviors, and depressive-like behaviors using a battery of behavioral tests. In the OFT (Fig. 2A), no differences were observed in the total distance traveled among groups, indicating that CSDS did not impair general locomotion and that behavioral alterations observed in the following assays are driven by emotional reactivity rather than motor deficits. The OFT further revealed a marked reduction in exploratory behavior and increased anxiety-like responses in SS mice. These animals exhibited fewer entries and spent less time in the center of the arena, consistent with heightened anxiety. RES mice showed intermediate behavioral profiles, with center entries and time partially restored toward CTRL levels. Anxiety-like behavior was further evaluated in the EPM (Fig. 2B). Although time spent in the open arms did not differ between groups, both SS and RES mice displayed a reduced number of risk assessment events. Notably, only SS mice exhibited a decrease in the duration of these behaviors, reinforcing the presence of an anxiety-like phenotype specifically in the SS group.

To assess depression-like behavior, we employed the TST (Fig. 2C). However, no significant differences in immobility time were observed, suggesting the absence of stress-induced despair-like behavior in this paradigm. To integrate behavioral outcomes across all tests, we computed z-scored group means for each behavioral variable and visualized them in a radar plot (Fig. 2D). This analysis highlighted the multidimensional emotional alterations in SS mice, with a pronounced anxiety-like profile and partial normalization in the RES group. These findings indicate that the CSDS paradigm successfully differentiates SS and RES phenotypes, with SS mice displaying robust anxiety-like behaviors across complementary paradigms.

### Resilience to CSDS is associated with reduced A2A receptor and excitatory synaptic markers in the NAc

**Fig. 3:**
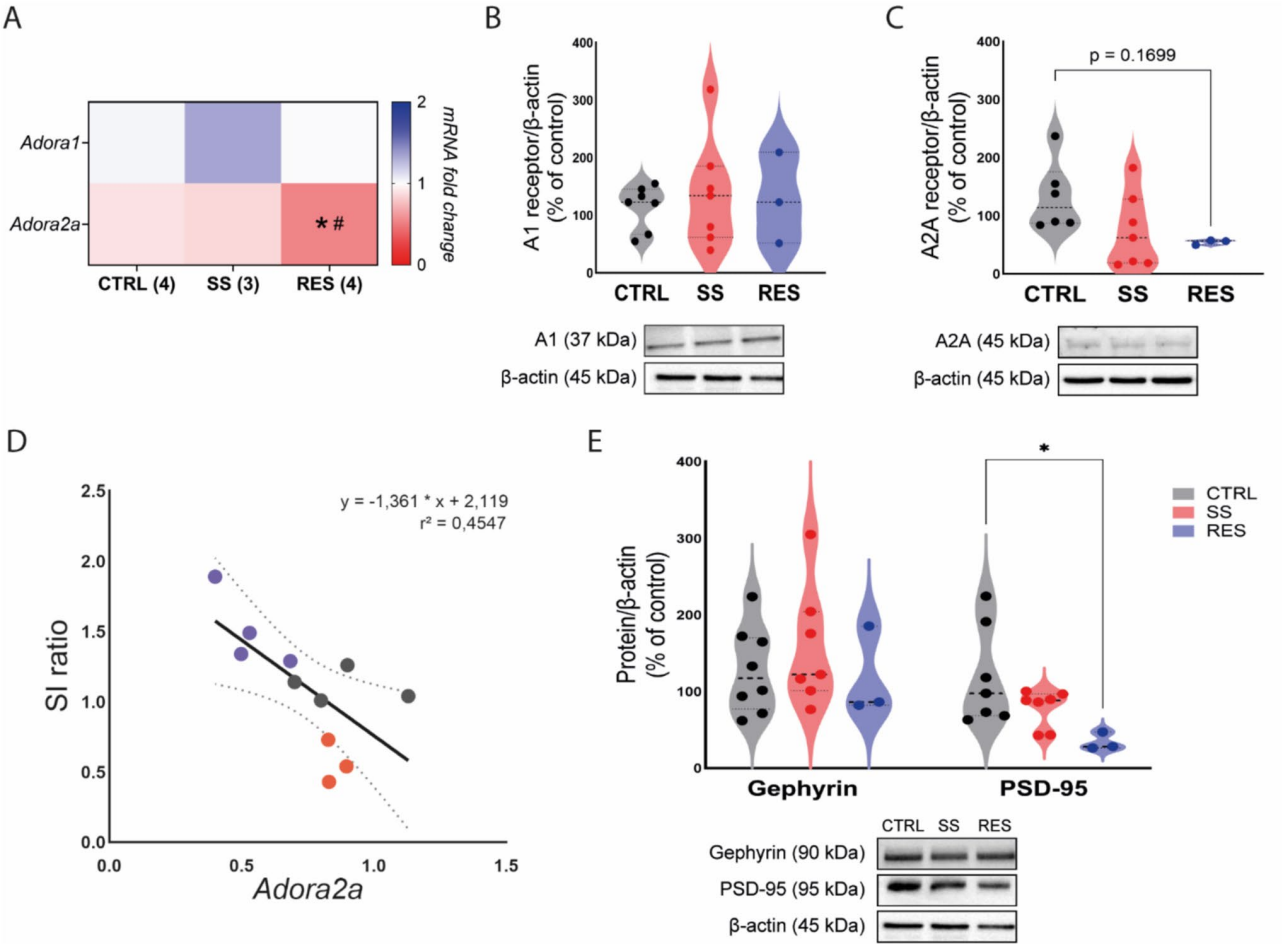
Stress resilience is associated with A2A receptor downregulation and diminished excitatory proteins. **A.** Heatmap of mRNA expression (2^ΔΔCt^) for *Adora1* and *Adora2a* in the NAc. *Adora2a* was downregulated in resilient (RES) mice compared with control (CTRL; *) and susceptible (SS; #). **B.** A1 receptor protein expression was not altered across groups. **C.** RES mice show a tendency of reduced A2A receptor protein levels. **D.** Linear regression showing a moderate negative correlation between *Adora2a* expression and social interaction (SI) ratio. **E.** Protein levels showing stable postsynaptic scaffolding proteins gephyrin (inhibitory synapses), but a reduction on PSD-95 of RES mice (excitatory synapses). One-way ANOVA followed by Tukey’s post hoc test (adenosine receptors genes and proteins), and Kruskal–Wallis with Dunn’s multiple comparisons (synaptic proteins). Data are shown as individual values, mean ± SEM, *, ^#^*p* < 0.05

To investigate purinergic mechanisms underlying SS and RES phenotypes, we assessed the expression of adenosine receptors A1 and A2A, along with key postsynaptic markers in the NAc, a region critically involved in reward processing and stress responsiveness. Analysis of *Adora1* mRNA expression revealed no significant differences between groups (Fig. 3A). Consistently, A1 receptor protein levels remained unchanged (Fig. 3B), suggesting a limited role for A1-mediated signaling in mediating stress-induced adaptations in this region.

In contrast, *Adora2a* mRNA expression was reduced in RES mice relative to CTRL (Fig. 3A), whereas protein levels did not differ between groups (Fig. 3C). This divergence between transcript and protein levels may reflect post-transcriptional mechanisms, suggesting that while *Adora2a* transcription is downregulated in resilience, protein stability or turnover could buffer changes at the receptor level. Linear regression analysis (Fig. 3D) aimed to determine the relationship between adenosine A2A receptor expression and behavioral outcomes. We observed a moderate negative correlation between Adora2a mRNA levels and the SI ratio, indicating that lower Adora2a transcript levels are associated with higher sociability and the RES phenotype; however, this relationship does not necessarily imply corresponding changes at the protein level.

Given the predominant postsynaptic localization of adenosine receptors and their modulatory role in glutamatergic and GABAergic neurotransmission, we next evaluated the expression of key excitatory and inhibitory postsynaptic proteins (Fig. 3E). Consistently, reduction of the excitatory postsynaptic marker PSD-95 was observed in RES mice, while levels of the inhibitory marker gephyrin remained unchanged. This reduction in excitatory postsynaptic markers aligns with the observed downregulation of gene expression of A2A receptors, suggesting a coordinated synaptic remodeling process associated with the RES phenotype.

### Chronic stress responses are predicted by caffeine sensitivity

**Fig. 4:**
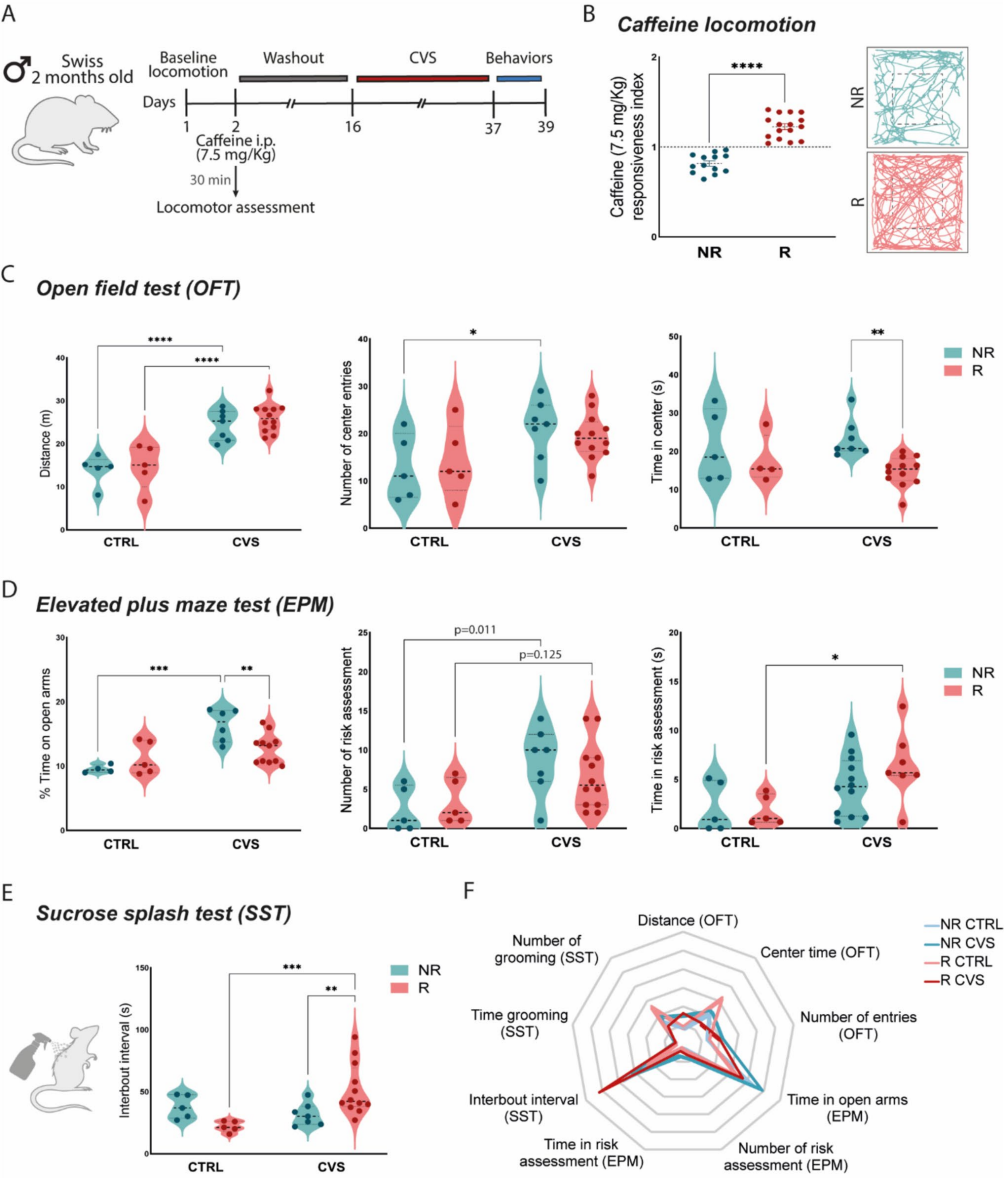
Caffeine responsiveness predicts behavioral outcomes for chronic variable stress (CVS). **A.** Experimental design: baseline locomotion was assessed, followed by caffeine responsiveness testing (7.5 mg/kg, i.p.), a 14-day washout, exposure to CVS, and behavioral assessments. **B.** Classification of mice as caffeine non-responsive (NR) or responsive (R) based on locomotor activity following caffeine administration, with representative locomotor traces. **C.** In the open field test (OFT), CVS increased locomotor activity in both groups (left). However, only NR mice displayed enhanced exploratory behavior, with increased center entries (middle) and time in the center (right). **D.** In the elevated plus maze (EPM), NR stressed mice spent more percentage of time in open arms (left), while R stressed mice showed prolonged risk assessment behavior (middle and right). **E.** In the sucrose splash test (SST), R mice subjected to CVS exhibited longer interbout intervals of grooming, indicating motivational deficits. **F.** Radar plot summarizing behavioral outcomes across all tests, with each axis representing one behavioral parameter. Welch’s T test (caffeine responsiveness), two-way ANOVA (OFT, EPM, and SST parameters). Data are shown as individual values, mean ± SEM, *p < 0.05, **p < 0.01, ***p < 0.001, ****p < 0.0001

We next assessed whether caffeine-induced hyperlocomotion could predict vulnerability or resilience to chronic stress, given its involvement in mood regulation via A2A receptor antagonism. For that, the CVS protocol was performed in 2-month-old male Swiss mice (Fig. 4A), a strain characterized for high behavioral heterogeneity and pronounced sensitivity to caffeine, responding to substantially lower stimulatory doses than common reference strains such as C57BL/6 (ED₅₀ ~ 4 mg/kg vs. ~ 18 mg/kg) [38, 39]. To test whether baseline adenosine signaling predicts stress vulnerability, animals were classified as caffeine-responsive (R) or non-responsive (NR) according to their locomotor activity following acute caffeine administration (Fig. 4B). R mice displayed a robust hyper locomotor response, confirming sensitivity to A2A receptor antagonism. After a washout period, animals were exposed to CVS, and emotional behavior was evaluated using a battery of tests.

In the OFT (Fig. 4C), both CVS groups exhibited increased locomotion; however, exploratory behaviors were differentially affected. NR mice subjected to CVS displayed more center entries and spent longer in the center, a pattern suggestive of disinhibited exploration. In contrast, R mice failed to show this increase, reflecting a distinct behavioral profile under stress. Anxiety-like behavior was further assessed in the EPM (Fig. 4D). NR mice exposed to CVS spent less time in the open arms compared to both R and CTRL counterparts, indicating heightened anxiety. While both CVS groups showed a tendency toward reduced numbers of risk assessment, only R mice demonstrated an increased duration of this behavior, suggesting persistent engagement in exploratory behavior despite stress exposure, which may reflect an altered strategy to cope with anxiogenic environments.

Depressive-like behavior was assessed with the sucrose splash test (Fig. 4E). R mice subjected to CVS exhibited longer interbout intervals, indicative of impaired motivational and self-care. This effect was absent in NR mice, further supporting a selective vulnerability of the R group to stress-induced motivational deficits. Integrated behavioral outcomes were summarized in a radar plot (Fig. 4F). This analysis revealed a multidimensional emotional profile, with R mice showing consistent anxiety- and depression-like features across OFT, EPM, and SST following CVS. Altogether, baseline caffeine responsiveness reliably predicts susceptibility to chronic stress, highlighting distinct emotional phenotypes.

## Discussion

Individual variability in stress responses is increasingly recognized as a determinant of mood disorder risk or resilience [40, 41]. While environmental factors, such as social experiences and lifestyle, shape stress outcomes, intrinsic neuromodulatory systems influence how organisms respond to adverse conditions [42]. Adenosine A2A receptor signaling in the NAc has emerged as a central regulator of excitatory synaptic drive and behavioral adaptation to chronic stress [24]. Here, we show that baseline differences in caffeine responsiveness, which reflects individual differences in adenosinergic tone, predict divergent behavioral trajectories following chronic stress exposure. Our findings link molecular and synaptic alterations in A2A receptor pathways to resilience or susceptibility, providing mechanistic insight into how neuromodulatory profiles modulate stress-related outcomes. We demonstrated that RES mice exposed to CSDS exhibit reduced *Adora2a* mRNA levels and decreased PSD-95 expression in the NAc, suggesting that dampened excitatory synaptic drive supports resilience. In contrast, SS mice maintained A2A receptor gene and protein levels, consistent with maladaptive excitatory signaling. In parallel, baseline caffeine sensitivity predicted outcomes in a CVS paradigm: caffeine-R mice displayed robust anxiety- and anhedonia-like behaviors, whereas NR animals exhibited a distinct profile characterized by altered exploratory strategies. Together, these findings indicate that A2A signaling is a critical determinant of stress vulnerability and resilience.

Our results align with studies demonstrating protective effects of reduced adenosine A2A receptor activity against stress. Genetic depletion or pharmacological antagonism of A2A receptors attenuates depressive- and anxiety-like behaviors in rodents [25, 43]. In humans, *Adora2a* polymorphisms and sensitivity to caffeine have been linked to anxiety traits [20, 21], further supporting the idea that interindividual variation in adenosine signaling contributes to psychiatric vulnerability [18, 44]. Importantly, our data extend these findings by showing that reduced A2A receptor expression in the NAc coincides with synaptic remodeling and resilient behavior, highlighting the role of local adenosinergic modulation in shaping stress outcomes.

The observation that RES animals display decreased PSD-95 expression in the NAc suggests that resilience involves reduced excitatory input to this region. This is consistent with previous reports showing that excessive excitatory drive from hippocampal and cortical inputs promotes susceptibility to social defeat, while dampening excitatory signaling fosters adaptation [9, 45, 46]. By coupling molecular changes in A2A receptor expression with alterations in excitatory synaptic markers, our results point to a coordinated mechanism that limits NAc overactivation under stress conditions. Conversely, SS animals may fail to engage these compensatory adaptations, leading to heightened vulnerability.

The behavioral divergence between caffeine-responsiveness further emphasizes the predictive value of adenosinergic tone. Indeed, the psychostimulant effect of caffeine depends on A2AR of NAc shell [47]. R mice, which displayed stronger baseline hyperlocomotor responses to caffeine, later developed motivational and anxiety-like deficits under CVS, suggesting that baseline A2A receptor sensitivity confers prediction for maladaptive emotional outcomes. In contrast, NR mice maintained different behavioral responses, marked by disinhibited exploration in the OFT but preserved motivation in depressive-like paradigms such as the SST. This dissociation highlights that stress vulnerability is not uniform but rather stratified according to intrinsic neuromodulatory profiles. The role of A2A receptors in modulating stress responses is further supported by studies indicating that selective A2A antagonists can attenuate depression-like behaviors in rodents, whereas overexpression of these receptors exacerbates such behaviors [23, 48]. For instance, administration of SCH 58261, a selective A2A receptor antagonist, reduced immobility time in the forced swim and tail suspension tests, suggesting antidepressant-like effects [49].

From a translational perspective, our findings suggest that A2A receptor antagonists could represent promising therapeutic strategies for stress-related mood and anxiety disorders. Indeed, istradefylline, a selective A2A receptor antagonist, has demonstrated antidepressant-like effects in animal models and has been shown to improve MDD symptoms, including anhedonia, in patients with Parkinson's disease [16, 50]. Moreover, the predictive role of baseline adenosine tone modulated by caffeine raises the possibility that treatment efficacy will vary across individuals. Caffeine has been shown to act differently in individuals depending on factors such as dose, genetic variations, and habitual intake [51]. While low-dose locomotor stimulation by caffeine is mainly mediated by A2A receptor antagonism, its non-selective blockade of both A1 and A2A receptors makes it difficult to attribute behavioral effects solely to A2A antagonism. Genetic polymorphisms in the *Adora2a* also contribute to individual sensitivity to caffeine effects on sleep [52]. Furthermore, habitual caffeine consumption has been associated with *Adora2a* mutations, with individuals with certain genotypes consuming more caffeine [53]. These interindividual differences highlight the complexity of caffeine's effects. Identifying biomarkers of adenosinergic function, such as caffeine responsiveness, may help to identify individual vulnerability to stressors and risk for psychiatric outcomes.

However, several limitations should be considered. First, our experiments were conducted exclusively in male mice, although sex differences in stress responses have been reported [54]. Second, while we focused on A1 and A2A receptors, additional purinergic targets, as well as interactions with dopamine and glutamate systems, may contribute to the observed phenotypes [14, 27]. Importantly, as immunoblottings of GPCRs such as A2A receptors are influenced by antibody specificity and the semi-quantitative nature of the technique, these findings should be interpreted within this methodological context. Finally, the discrepancy between *Adora2a* mRNA and protein levels suggests that further work using functional assays is needed to fully characterize A2A receptor regulation under stress exposure [55]. Altogether, this study identifies reduced A2AR signaling and excitatory synaptic remodeling in the NAc as hallmarks of resilience, while heightened caffeine sensitivity predicts vulnerability to chronic stress. These findings advance our understanding of the adenosinergic system as a mediator of stress heterogeneity and support its potential as a therapeutic target for mood disorders.

## Conclusion

Our results show that adenosine A2A receptors in the NAc and baseline caffeine responsiveness are possible determinants of resilience or vulnerability to chronic stress. Reduced A2AR expression and excitatory synaptic remodeling promote adaptation, whereas heightened A2AR sensitivity predisposes to anxiety- and anhedonia-like behaviors. These findings highlight adenosinergic signaling as a promising therapeutic target and suggest that interindividual variation in caffeine responsiveness may serve as a translational biomarker in stress-related disorders.

## Data Availability

Data will be made available upon request.
